# Influence of motor imagery training on hip abductor muscle strength and bilateral transfer effect

**DOI:** 10.3389/fphys.2023.1188658

**Published:** 2023-09-07

**Authors:** Majid Manawer Alenezi, Amy Hayes, Gavin P. Lawrence, Hans-Peter Kubis

**Affiliations:** ^1^ Department of Sport and Exercise Sciences, School of Human and Behavioural Sciences, Bangor University, Bangor, United Kingdom; ^2^ Northern Border Health Cluster, Academic Affairs and Training, Arar, Saudi Arabia

**Keywords:** motor imagery, mental training, musculoskeletal muscle, exercise, motor function, rehabilitation

## Abstract

Motor imagery training could be an important treatment of reduced muscle function in patients and injured athletes. In this study, we investigated the efficacy of imagery training on maximal force production in a larger muscle group (hip abductors) and potential bilateral transfer effects. Healthy participants (*n* = 77) took part in two experimental studies using two imagery protocols (∼30 min/day, 5 days/week for 2 weeks) compared either with no practice (study 1), or with isometric exercise training (study 2). Maximal hip abduction isometric torque, electromyography amplitudes (trained and untrained limbs), handgrip strength, right shoulder abduction (strength and electromyography), and imagery capability were measured before and after the intervention. Post intervention, motor imagery groups of both studies exhibited significant increase in hip abductors strength (∼8%, trained side) and improved imagery capability. Further results showed that imagery training induced bilateral transfer effects on muscle strength and electromyography amplitude of hip abductors. Motor imagery training was effective in creating functional improvements in limb muscles of trained and untrained sides.

## Introduction

Motor imagery (MI) is a cognitive simulation technique. It is defined as a mental process whereby an individual rehearses or simulates a specific motor actions/task (Guillot and Collet, 2008). MI represents one of the most widely used cognitive interventions to enhance both sports (Sordoni et al., 2000; Guillot and Collet, 2008; Monsma et al., 2009; Wang et al., 2014) and therapeutic performance (Kaschel et al., 2002; Morganti et al., 2003; Liu et al., 2004; Bovend’Eerdt et al., 2010; Heremans et al., 2011; Hussey et al., 2012; Braun et al., 2013; Caligiore et al., 2017; Ruffino et al., 2017) and the last 2 decades has seen a considerable amount of research advocating MI as an effective rehabilitation tool for motor function restoration, i.e., in stroke patients. Therapeutic effects of MI were detected for enhancing motor facilitation and gait relearning, and improvements in distinct motor functions in various conditions (stroke, Parkinson’s disease, brain injury) (Mulder, 2007; Zimmermann-Schlatter et al., 2008; Braun et al., 2013).

MI can be engaged in via visual (with internal, or first person, and external, or third person, perspectives), kinaesthetic (based on somatosensory information), and tactile modalities (Hardy and Callow, 1999; Roberts et al., 2008; Kim et al., 2011; Ruffino et al., 2017). Previous studies investigating the effect of MI interventions on muscle strength have adopted the sole use of MI without any physical execution and typically without any consideration of imagery modalities or perspectives (Ranganathan et al., 2004; Reiser, 2011; Reiman et al., 2012); for exception see (Herbert et al., 1998). Moreover, effectiveness of MI on muscle strength is shown to be influenced by factors like duration of training, type of muscle groups, and age of participants (Paravlic et al., 2018; Liu et al., 2023).

Possible explanations for the effective nature of MI reside in increases in muscle strength and function (Herbert et al., 1998; Paravlic et al., 2018; Liu et al., 2023). However, most of the research reporting these effects focus on relatively small muscle groups (i.e., upper limb muscle) with variable effectiveness reported when larger muscle groups (lower limb muscles) are targeted for treatment (Ranganathan et al., 2004; Yao et al., 2013; Grosprêtre et al., 2018; Bouguetoch et al., 2021). The underlying mechanism of MI training on muscle strength is believed to be based on neural adaptation. Specifically, it is shown that MI leads to elevation of Movement-Related Cortical Potential (MRCP) over both the primary motor and supplementary motor cortices (Herbert et al., 1998; Shackell and Standing, 2007). Here, it is suggested that MI strengthens the brain-to-muscle command processes and improves both motor unit recruitment and activation, leading to greater muscle forces (Shackell and Standing, 2007). While most of the literature reports an increase of brain activations (measured via evoked potentials and changes in blood flow (fMRI)) when comparing MI with rest (i.e., an online effect), Grosprêtre et al. (2018) reported that MI practice can influence cortical descending neural drive (Grosprêtre et al., 2018). Here, 1 week of daily MI practice resulted in an increase of the supraspinal command and spinal network excitability which may explain the gains observed in maximal force and rate of torque development. Moreover, MI may lead to a more efficient cortical drive to motor units leading to reduced agonist/antagonist coactivation with increased force performance of the targeted muscle group (Dos Anjos et al., 2022).

Interestingly, the MI effects on large muscle group strength (lower limb) and function have primarily focused on ipsilateral based paradigms, i.e., the effects of MI on the imagined limb/muscle group (Paravlic et al., 2018; Liu et al., 2023). Somewhat counter intuitively, in physical training interventions the effects of exercise training on the trained muscle group (ipsilateral training effect), can also have effects on the contralateral untrained muscle group (Munn et al., 2004). This cross-education effect, is an inter-limb phenomenon and has been extensively investigated (Lee and Carroll, 2007; Land et al., 2016; Manca et al., 2021). It is defined as the transfer effect from the trained limb to the homologous contralateral untrained limb following unilateral training (Farthing et al., 2007). However, this effect is rarely investigated in the context of MI (Land et al., 2016). Moreover, research has yet to investigate if the contralateral transfer effect can be observed in homologous muscle groups other segments (i.e., transfer from the leg muscle group to the homologous arm muscle group). Moreover, current understanding of physiological responses to MI training centres on specific responses in the muscles involved in the imagined movement, rather than any form of generalized arousal response (Lang, 1979; Zijdewind et al., 2003). Investigating the bilateral transfer effect with MI training in muscle groups with particular relevance to sport rehabilitation and physiotherapy practice could provide an important foundation for the future application of MI training improving the efficacy of rehabilitation protocols (Dickstein and Deutsch, 2007; Arora et al., 2011). For example, MI training could be used at different stages of rehabilitation protocols in connection with physical training or as sole intervention when physical training is unattainable due to pain, compliance, or medical barriers (Sidaway et al., 2005; Guillot and Collet, 2008; Dickstein et al., 2013).

We performed two studies; study 1 examined the efficacy of two different imagery modalities in comparison to control on hip abductor muscle strength. Specifically, participants adopted either kinaesthetic imagery (KIN), KIN imagery in conjunction with visual imagery (KIN + VI) or control (no imagery training). In study 2 we examined the efficacy of imagery training in comparison to isometric exercise training. This study used the more effective imagery training intervention from the results of study 1; i.e., (KIN + VI). The overall aims of the research were 1) to examine the ipsilateral training effects of different imagery modalities on large muscle groups, 2) to exploring the bilateral transfer effect in strength and EMG outcomes following unilateral imagery and exercise training 3) to investigate transfer effects of imagery and exercise training to a different body segment to that targeted by the imagery and training interventions (i.e., transfer to analogous functional muscle groups).

For both studies, the large muscle group of the gluteus medius muscle was targeted for intervention because it is of clinical importance in physiotherapy (Gowda et al., 2014) and essential function in athletic pursuits (Kumagai et al., 1997; Reiman et al., 2012). The muscle works as a primary abductor of the hip joint, is an essential muscle during walking, and works as a pelvis stabiliser in a unilateral stance against gravity (Kumagai et al., 1997).

In study 1 we hypothesized that combined KIN + VI imagery training would be more effective than KIN alone and result in significantly greater gains in hip abductor muscle strength. In study 2, we hypothesized that training with combined + VI imagery would lead to significant increases in maximal isometric force production of hip abductors and induce a bilateral transfer effect similar to exercise training.

## Material and methods

### Study 1

#### Experimental approach to the problem

To test the authors’ hypothesis, we used a randomized control design with three arms, i.e., participants were randomly assigned to one of either of the two training groups or the control group. This design enabled authors to explore the feasibility and superiority of distinct imagery modalities compared with no training group.

#### Participants

After study approval by the research ethics committee of the School of Human and Behavioural Sciences, Bangor University, UK, fifty-one participants (26 women), mean age = 24.43, SD = 5.75) volunteered to participate in the randomized controlled trial and provided written informed consent. All participants were naïve to the experiment hypothesis, free from recent lower limb injuries, and able to perform physical assessment activity. Forty-seven participants completed the study and were included in the analysis; baseline scores of body characteristics) showed no significant differences between groups (see Table 1). Participants were reimbursed for their time (£50 for experimental groups and £20 for the control group so that participants were approximately equally compensated for their time investment according to recommendation by Breitkopf et al. (2011). Participants were recruited with knowledge that they would be reimbursed for the time actively partaking in the study. At the end of the study, participants received the reimbursement according to the difference in time.

**TABLE 1:** Demographic characteristics of the study groups.

Parameter	KIN + VI (n = 16)		KIN (n = 16)		CTRL (n = 15)	
	M	SD	M	SD	M	SD
**Age (yrs)**	24.06	4.95	24.00	5.69	26.20	6.97
**Gender (# women)**	11		7		5	
**Height (cm)**	172.42	7.36	174.47	8.28	175.70	7.45
**Mass (kg)**	68.49	9.46	72.81	8.05	74.91	5.72
**BMI (kg/m** ^**2**^)	22.94	1.79	23.94	2.41	24.36	2.56
**Physical activity**	1.94	0.57	2.06	0.44	2.20	0.77

The categories of physical activity scores 1 = low, 2 = moderate, 3 = high activity. M, mean value; SD, standard deviation.

#### Imagery intervention

**FIGURE 1:**
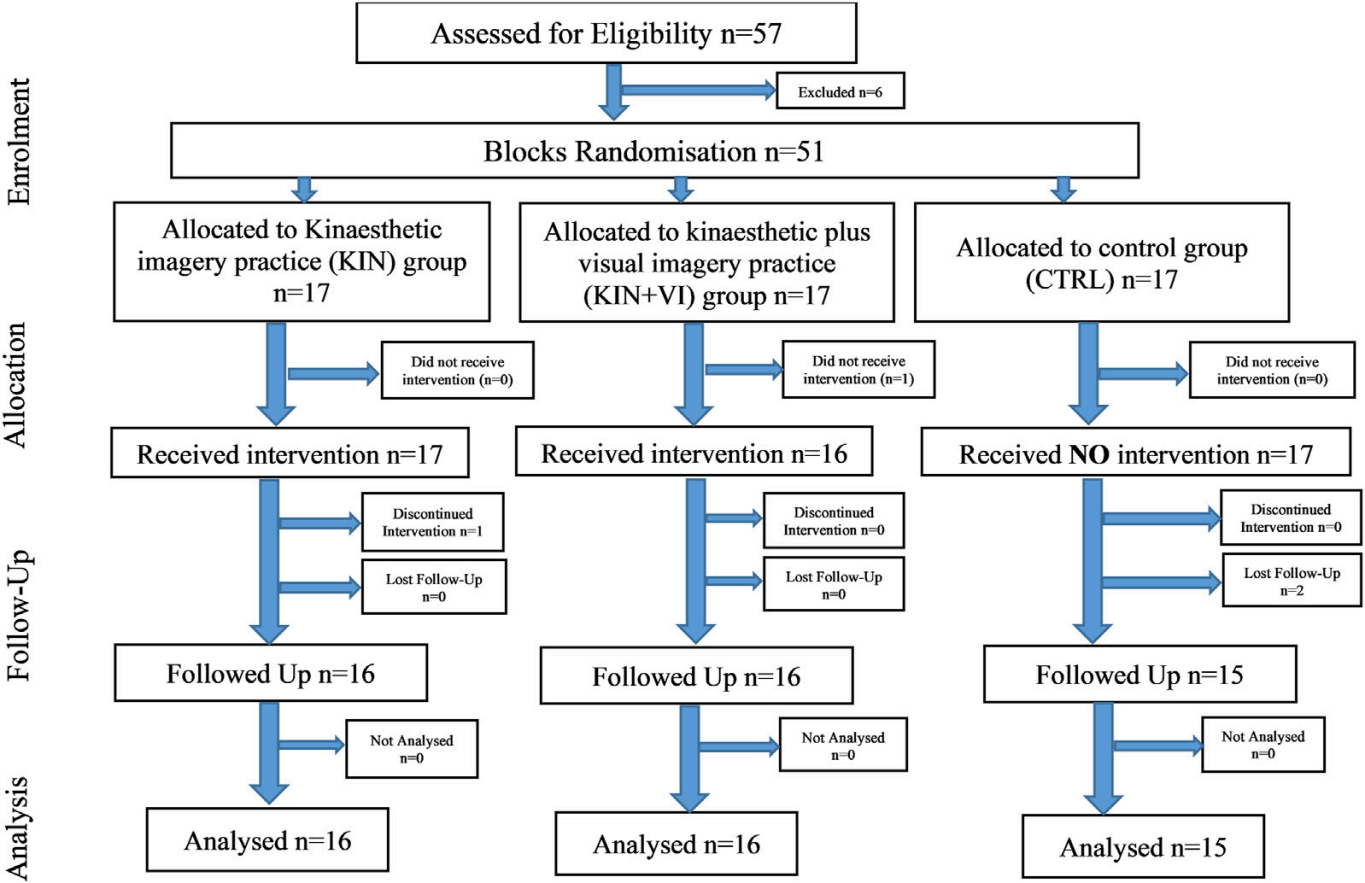
Study 1 outline.

Two imagery protocols were used in the experimental groups. The intervention protocols comprised of mentally simulating (imagined maximal isometric contractions of hip abductors) the physical task (push against dynamometer arm) used to assess hip abductors strength without any actual physical execution. One protocol used KIN modality alone and the other combined KIN + VI: (S1 Protocol). Participants were randomly allocated to either one of the imagery intervention groups (KIN group (n = 16); KIN + VI group (*n* = 16)) **or a no practice** control group (CTRL; n = 15; one dropout) (see Figure 1).

Following random allocation to groups, all participants undertook two pre-test intervention sessions. In the first, participants completed a Health Questionnaire, the Vividness of Movement imagery Questionnaire-2 (VMIQ-2) (Roberts et al., 2008), and the International Physical Activity Questionnaire (IPAQ) (Craig et al., 2003). In the second, participants performed a strength test; a maximal isometric torque (MIT) assessment of right hip abductors on a dynamometer (see below). Subsequently, the schedule for daily imagery training visits was produced. One familiarization session for each participant in the imagery groups was performed (∼30 min session), where they received instructions and training on how to perform the distinct imagery exercises protocol. Participants also received written information explaining the training protocol and the specific imagery script of their group. This was followed by an intervention period of 2 weeks, during which participants of the two intervention groups had five imagery training sessions per week (10 in total). The duration of each session was about 30 min; each participant imagined the maximum isometric contraction of right hip abductors for 35 times over seven sets of imagery training (S1 Protocol).

Participants in the experimental groups completed the imagery practice individually with the experimenter in a quiet room. Participants were given an imagery script specific to their imagery group. The scripts reflected Lang’s (1979) recommendations that imagery scripts be based on the imager’s own experiences, and that the imagery include stimulus propositions (i.e., physical details of objects), response propositions (e.g., muscle tension), and meaning propositions (e.g., interpretation of the image) (Lang, 1979; Callow et al., 2013). The scripts instructed participants to remember their experience of producing the maximal muscle contraction during the pre-intervention MIT strength test (Reiser, 2011). The KIN group’s script instructed participants to, imagine that you are lying on your side on the isokinetic machine. Think back to your own sensations you felt as you performed your maximal contraction of your thigh. Feel your top leg rise to meet the fixed dynamometer arm. Feel the cushion of the dynamometer touch the side of your thigh just laterally above the knee…. The KIN + VI group’s script was exactly analogous to the KIN script, except with the additional instruction to use visual imagery, imagine that you are lying on your side on the isokinetic machine. Think back to the sensations you felt and your own visual image as you performed your maximal contraction of your thigh. The order in which kinaesthetic or visual imagery instructions were presented in the KIN + VI group’s scripts was counterbalanced across participants. KIN + VI participants were not instructed regarding which visual perspective, internal or external, to use. While there is evidence that imagery from an internal, or first-person, visual perspective tends to be more effective for open skill tasks requiring responses to objects (Reiser, 2005), and external (third-person) perspective is more effective for tasks such as gymnastics that require specific form (Hardy and Callow, 1999), it is not clear whether open skill or form best describes the muscle contraction task. Therefore, participants were free to use either perspective. The scripts were presented as audio recordings and in written form. In each session participants imaged the muscle contraction 40 times for 15 s each. Participants were instructed not to perform actual muscle contractions during the imagery. Compliance was monitored by the experimenter visually observing the participant for any visible physical contraction during imagined contractions (Reiser, 2005; Reiser, 2011); participants were informed if any contractions were seen and instructed to let their muscle relax during imagined contraction. Participants performed the mental practice with eyes closed. The control group did not receive any intervention during the 2 weeks.

All imagery sessions were delivered one-to-one by the principal researcher in a quiet research laboratory, which was also used for pre and post intervention assessments. Following the intervention, all participants were tested again for MIT and completed the VMIQ-2 questionnaire and post-experimental questionnaires.

## Measurements

### Isometric torque measurement

Measurement of MIT in right hip abductor muscles was performed using a Humac dynamometer (HUMAC Cybex NORM 2004; Computer Sports Medicine Inc., Stoughton, MA, United States) and analyzed using AcqKnowledge software (BIOPAC Systems Inc., Goleta, CA, United States). All tests were ‘make’ tests where the dynamometer and participants were positioned by the examiner to exert a maximum force against the dynamometer lever. Test positions were standardized for the hip abductors (Jan et al., 2004; Reiman et al., 2012). Participants were asked to perform a 5-min warm-up on a cycle ergometer prior to the MIT assessment. Each participant then completed four submaximal contractions as familiarisation followed by 2 min of rest. Three test trials for right hip abductors were then performed. Each test trial lasted for approximately 5 s to allow the participant to generate maximum voluntary effort. Each of the three test trials were separated by 1-min to allow for rest and recovery. The trial with the maximum force generated (Nm) was recorded as the participants MIT value (Jan et al., 2004).

### Imagery ability

The Vividness of Movement Imagery Questionnaire-2 (VMIQ-2) was administered to assess the participants’ imagery ability (Roberts et al., 2008). The VMIQ-2 is a suitable psychometric measurement of movement imagery ability with acceptable factorial, concurrent, and construct validity (Roberts et al., 2008). The questionnaire assesses imagery ability for three separate modalities, internal visual imagery (IVI; i.e., visual imagery from a first-person perspective), external visual imagery (EVI; i.e., imagining watching oneself perform an action from a third-person perspective), and kinaesthetic imagery (i.e., imaging the sensation and feeling of performing an action or movement, including the force, effort, balance, and spatial location associated with the movement). The questionnaire is comprised of 12 items that assess imagery ability for a variety of movements (e.g., running upstairs, kicking a stone). Each of the 12 imaged items is rated separately for each of the three imagery modalities using a 5-point Likert scale with values from 1 (*perfectly clear and vivid*) to 5 (*no image at all; you only know that you are “thinking” of the skill*).

### Motivation and effort questions

Motivation to perform the strength task and subsequent effort applied in performing the task were assessed via questionnaire at the pre and post assessment sessions. The level of motivation during performing the strength task was assessed by using a Likert scale to answer the question “How motivated were you to succeed in that task?” with zero representing “Not motivated at all” and 10 representing “very highly motivated”. The level of effort in performing the strength task was assessed by a numerical scale (0–150), where zero represents “*No* effort at all” and “110” and above represents “*Extreme* effort”.

Twenty participants across the study groups were assessed pre and post. Both measures were assessed due to a potential influence of motivation and effort investment on strength outcomes.

### The International Physical Activity Questionnaire (IPAQ)

The IPAQ was administered at pre-test level and assesses individual’s physical activity level (Craig et al., 2003).

### Post intervention questionnaire

Following the intervention period, participants were asked three questions to assess the intensity of using specific imagery modalities. These data were used to assess participants’ commitment to implement the use of specific imagery modalities. Participants’ usage of KIN and VI modalities were graded using a Likert scale, zero representing (No KIN or VI use) and 10 representing (High KIN or VI use). Further questions were used to investigate which VI perspective (internal IVI)—external (EVI)) was adopted while using VI; graded on a Likert scale, zero representing (Completely IVI), 5 representing (Switched regularly), and 10 representing (Completely EVI).

### Statistics

Statistical analyses were performed in SPSS software for Windows (version 27.0; IBM Corp, Armonk, NY, United States). Data sets were normally distributed according to Shapiro-Wilk test. Baseline and post intervention variables of questionnaires were assessed between groups by one-way Analysis of Variance (ANOVA). The main outcomes MIT and VMIQ-2 scores were assessed using a one way between group ANOVA on the model of change data (i.e., post-minus pre-test). A *post hoc* test with Bonferroni adjustment was performed for multiple comparison purposes. A significance level of *p <* 0.05 was considered significant. The MIT and VMIQ-2 data were analysed by ANOVA of change due to significant difference of baseline levels between groups at pre-assessment. This method has been shown to produce less bias in the analysis than ANCOVA when pre-test differences between groups are large (Van Breukelen, 2006). Data are presented as mean and standard deviation if not mentioned otherwise. The original sample size was chosen in relation to logistical reasons, i.e., limitation in recruitment success. However, for this study, a *post hoc* power calculation (G*Power 3.1.9.7) was performed for repeated measures ANOVA within-between interaction based on the interaction effect size of MIT (f = 0.449), α error = 0.05, n = 47, and three groups, two measurements. The achieved power was (1-β) = 0.99 showing that the study was not underpowered.

### Study 2

#### Experimental approach to the problem

This study was designed using a randomized control design with two arms, imagery, and exercise. The design was aimed to investigate the efficacy of the imagery protocol (i.e., the more effective protocol from study one) compared with an isometric exercise protocol on muscle strength. In addition, this design was used to explore the underlying mechanism of imagery training by contrasting it to exercise training and investigating physiological effects on trained and untrained sides.

#### Participants

After ethical approval by the research ethics committee of the School of Health and Behavioural Sciences, Bangor University, UK, 33 healthy male participants (mean age = 25.50, SD = 3.99) volunteered to participate in the randomized controlled trial and provided informed written consent. All were healthy, right-handed, males, free from recent lower limb injuries, and able to perform physical assessment. Based on study 1 outcomes for MIT, an *a priori* sample size was calculated (G*Power 3.1.9.7) for a repeated measure ANOVA, within-between interactions using the interaction effect f = 0.449, α error = 0.05, Power (1-β) = 0.95, 2 groups, 2 measurements, which gave a sample size of at least 20 participants. Data from 30 participants who completed all study procedures were included in the analysis; body characteristics at baseline revealed no significant differences between groups (Table 2). Participants were reimbursed £50 for their time.

**TABLE 2:** Demographic data of all participants across study groups.

Parameter	*Exercise* (n = 15) M SD		*Imagery* (n = 15) M SD	
	M	SD	M	SD
**Age (yrs)**	26.53	4.22	24.47	3.58
**Height (cm)**	176.57	4.56	177.00	4.19
**Mass (kg)**	77.39	8.19	73.82	12.21
**BMI (kg/m** ^**2**^ **)**	23.48	3.16	23.55	3.73
**Physical activity**	2.40	0.51	2.47	0.52

The categories of physical activity scores are 1 = low activity, 2 = moderate activity, 3 = high activity. BMI, body mass index; M, mean value; SD, standard deviation.

#### Intervention protocols

Participants were randomized into two groups (*Imagery* and *Exercise*). Participants in the *Imagery* group mentally practised isometric contractions of the right hip abductors (ipsilateral training) with the KIN + VI script used in study 1. Participants in the *Exercise* group received a physical practice protocol involving “isometric exercise training” of right hip abductors. The training protocols of both the imagery and exercise group consisted of 10 supervised training sessions in a research laboratory over a 2-week period with sessions occurring on weekdays (Monday to Friday) (S1 Protocol); additional independent home practice was performed at the weekends.

The home training was mandatory for all participants and was facilitated using written and audio intervention scripts. A weekend diary log and a stopwatch were provided for each participant to facilitate performance and compliance with the home training protocol. The home protocols were structured according to the lab-supervised protocols (S1 Protocol).

### Study outline

**FIGURE 2:**
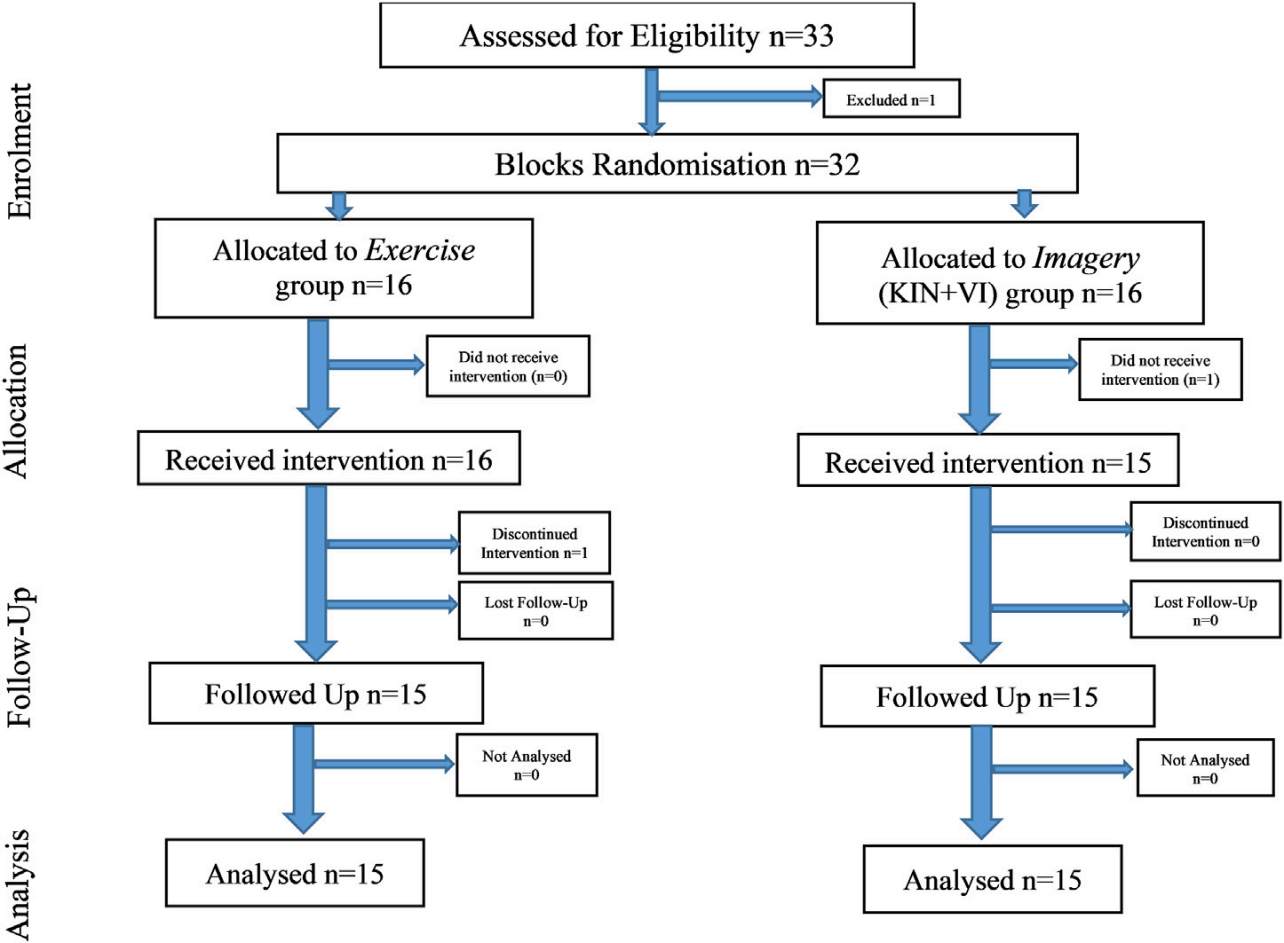
Study 2 outline.

Participants signed informed consent and were screened for study criteria. Selected participants were then randomly allocated to one of the two study groups, *Imagery* group (n = 15) or *Exercise* group (n = 15) (Figure 2). All participants undertook the pre-training session, which included familiarization with study procedures and measurements, completing a health questionnaire, IPAQ questionnaire, VMIQ-2 questionnaire, assessing demographic data, body characteristics, and performing practice trials for strength assessment on the dynamometer (as described in study one). The baseline assessment session consisted of performing left-hand grip strength together with the MIT assessment for the bilateral hip abductors, and right shoulder abductors muscle groups. In addition, EMG was performed on bilateral gluteus medius (GM) and right deltoideus medius muscles (R-DM). The MIT assessment was followed by the pre-intervention motivation and effort questions (see below) and participants were provided with information regarding each intervention type. Specifically, explanations of how to perform the assigned intervention protocols, e.g., maximal physical isometric contractions or imagined maximal isometric contractions. Moreover, participants received a written copy of the training protocol that included specific instructions regarding the intervention training (i.e., *Imagery* or *Exercise*) and practice on how to perform the specific training protocols. This was followed by an intervention period of 2 weeks. Here, participants had five training sessions per week (10 in total). The duration of each session was 30 min and each participant imagined/or performed the maximum isometric contractions of right hip abductors a total of 35 times over seven blocks of training (i.e., 5 per block). During the second intervention session, EMG amplitudes were recorded of the right hip abductor muscle (i.e., GM). In addition, the home programme was introduced to each participant which they were asked to perform on the weekend days (i.e., when experiment supervision was not present) and to complete the weekend diary log(S1 Protocol).

All imagery/exercise sessions were delivered as described for study 1. Following the training intervention, all participants were assessed again for MIT, EMG and completed the VMIQ-2 questionnaire, motivation and effort questionnaire, and a post-experimental questionnaire.

## Measurements

MIT was assessed for both hip abductor muscles and deltoid shoulder muscles (one side), surface EMG for shoulder and hip abductors, left handgrip strength, and participants imagery ability at baseline and after 2 weeks of intervention.

### Isometric torque measurement

The test procedure consisted of measurement of MIT in bilateral hip abductor muscles, using the same procedures, dynamometer, and protocols as described for study 1, except the order of side selection was randomized in a counter-balanced manner, left or right side first. In addition, after the first set of assessments, participants completed a 3-min cycling warm-up to avoid order effects and influences of hip abduction testing on the contralateral side.

Additionally, the test procedure involved measuring the MIT of right shoulder abductors with similar procedures and protocols that described in study 1. The settings for the chair and dynamometer differed in the test position, standardized for the shoulder abductors at 45° of abduction; participant position in the dynamometer followed the guidelines of the HUMAC dynamometer instructions.

### Handgrip strength measurement

The handgrip strength (HGS) of the left hand was measured using a Jamar Analogue Hand Dynamometer (Lafayette Instrument Company, Lafayette, IN, United States). Protocol comprised of one familiarization trial, then participants were asked to perform three maximal contractions and the mean used in the analysis (Roberts et al., 2011).

### Surface electromyography

Skin preparation included shaving, abrading, and cleaning the skin with 70% ethanol for controlling the inter-electrode resistance to be <5 Ω (Bolgla and Uhl, 2005). Consistent with SENIAM project (Surface Electromyography for the Non-Invasive Assessment of Muscles) recommendations, two silver/silver chloride surface (Ag/AgCl) electrodes (Neuroline 720 silver/silver chloride, Ambu, Ballerup, Denmark) were placed in a bi-polar configuration parallel to muscle fiber direction of the GM muscle and the DM muscle, with a center-to-center inter-electrode distance of 20 mm. Reference electrodes were placed on the wrist or on/around the ankle.

EMG signals were amplified with a bandwidth frequency ranging from 10 Hz to 500 Hz, then digitalized online at a sampling frequency of 2 kHz and recorded by the BIOPAC system (MP150, BIOPAC Systems Inc., Goleta, CA, United States), rectified, and stored with the force signal on computer disc. The positions of the EMG electrodes were marked on transparent sheets together with the location of the participant’s specific skin marks (e.g., birthmarks) to allow the electrodes to be placed in the same position for the post-intervention tests. Surface EMG recordings were collected simultaneously with the MIT assessments of the hip and shoulder abductors on the HUMAC dynamometer according to previous protocol. Raw EMG data of the bilateral GM and the R-DM muscle trials were obtained from EMG traces of three maximal isometric contraction trials. Each maximal contraction was held for 5 seconds with 1 minute of rest between each trial. All participants received an equal amount of verbal encouragement during data collection.

The average of the EMG’s Root Mean Square (RMS) that was associated with the highest peak force contraction was used for statistical analysis. The mean RMS values were identified by taking an average between 0.5 s before the peak force and 0.5 s after the peak force using magnified vison tools of the software identifying the start and finish of RMS. The EMG amplitude output was recorded in millivolts (mV).

### Self-reported assessment

The following questionnaires from study 1 were used: International Physical Activity, VMIQ-2, and Post-Experimental.

### Motivational and effort questionnaire

Participants level of motivation was assessed using a Likert scale to answer the question (How motivated were you to succeed in that task?) with zero representing (Not motivated at all) and 10 representing (very highly motivated). The level of effort was assessed using the Rating Scale of Mental Effort a numerical scale (0–150), where zero represents (*No* effort at all) and “110” and above represents (*Extreme* effort) (Rodgers et al., 1991). The levels of motivation and effort were included in this study for assessing their potential contribution to subsequent strength gain following the intervention trainings, either *Imagery* or *Exercise*.

### Weekend diary log

The weekend diary log assessed participants’ commitment to the home programme and levels of motivation after performing the home training sessions. The level of participants’ motivation to perform the home-sessions was assessed using a Likert scale with 0 meaning (Not at all motivated) and 10 representing (Highly motivated).

## Statistics

Baseline analysis across all study variables were completed. Muscle strength outcomes and imagery (VMIQ-2 subscales) were analysed using independent samples *t*-tests and EMG data using Mann-Whitney U test because data are not normal distributed. To assess the efficacy of the training interventions, sperate mixed model repeated measure (2 group x 2 time) Analysis of Variance (ANOVA) was used to analyse MIT, level of motivation, effort, and total VMIQ-2 score. Significant main effects and interactions were broken down using within group analysis (paired-samples *t*-tests).

EMG amplitude data (mV) were assessed using the Wilcoxon signed-rank test and a Mann-Whitney U test. All values are reported in means ± SD, except the EMG data, which are reported in median (50th) plus percentiles (25th, and 75th). A statistical level of *p <* 0.05 was deemed significant. All statistical procedures were performed using Statistical Package for Social Sciences (IBM SPSS) version 27.0 (IBM Corp., Armonk, NY, United States).

## Results

### Study 1

**FIGURE 3:**
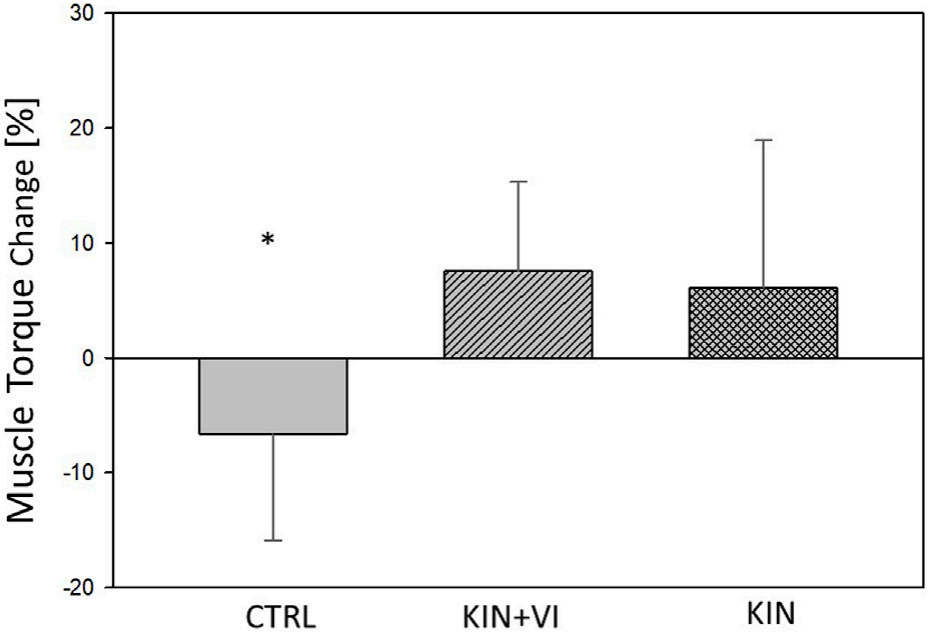
Changes in hip muscle strength pre to post training in study groups. CTRL: control group; KIN + VI: combined kinaesthetic imagery with visual imagery training; KIN: kinaesthetic imagery training. Mean and standard errors; *significant difference, *p* < 0.05.

Maximal isometric torque (MIT) of right hip abductors are listed in Table 3. After the 2 weeks imagery training, the ANOVA model revealed a statistically significant difference between study groups in muscle strength alterations (*F* (2.44) = 8.867, *p* = .001, η^2^ = 0.32) (Figure 3). The *post hoc* test revealed that strength alterations differed significantly in the KIN + VI (7.52 ± 7.80 Nm, *p* = .001) and KIN (6.08 ± 12.86 Nm, *p* = .003) compared with the CTRL group (−6.61 ± 9.29 Nm). There were no significant differences in strength change between KIN + VI and KIN groups (*p* = 0.92).

**TABLE 3:** MIT of right hip abductors across both study groups at pre- and post-assessments.

Study groups	MIT (Nm) right hip abductors	
	Baseline Strength	Post Strength
**KIN + VI (n = 16)**	120.70 ± 38.37	128.75 ± 36.15
**KIN (n = 16)**	126.06 ± 39.62	132.94 ± 41.44
**CTRL (n = 15)**	141.70 ± 44.25	132.29 ± 42.85*

*significant difference between groups on MIT, change (see text).

Analysis of the data for pre-post changes in motivation to perform and subsequent effort into the performance of the strength task (Table.4) revealed that there was a significant difference between groups in change of motivation pre to post (F (2.17) = 4.04, *p* = 0.037, η^2^ = 0.32), with *post hoc* Bonferroni test showing a significant difference between CTRL and KIN only (*p* = 0.036), with higher increase in motivation in the KIN group. Correlation analysis across all groups revealed a low correlation between changes in motivation and changes (%) in strength (R = 0.308, *p* = 0.200). Moreover, analysis of pre-post changes in effort performing the strength task revealed no significant change in effort between groups (F (2.17) = 0.764, *p* = 0.481). In addition, no significant correlation between effort change and strength change (%) across all groups were observed (R = −0.091).

**TABLE 4:** Levels of motivation and effort.

Parameter	KIN + VI (n = 6)		KIN (n = 7)		CTRL (n = 7)	
	Pre	Post	Pre	Post	Pre	Post
**Effort level** (zero: *no* effort at all, to 130: *extreme* effort)	108.3 ± 9.3	107.5 ± 7.4	106.1 ± 23.6	112.4 ± 11.5	102.9 ± 10.7	108.2 ± 18.8
**Motivation level** (zero: not motivated at all, to 10: very highly motivated)	9.17 ± 0.75	9.67 ± 0.82	8.43 ± 1.40	9.57 ± 0.79	8.29 ± 1.38	7.71 ± 1.89

In addition, ANOVA revealed a statistically significant alteration between study groups in VMIQ-2 score (% changes) (*F* (2.44) = 3.405, *p* = .042). Post-hoc test revealed that the VMIQ-2 change marginally differed in the KIN + VI (−7.99 ± 20.62) compared with KIN (7.97 ± 18.57, *p* = .089) and with CTRL group (9.54 ± 23.37, *p* = .061). There was no difference between CTRL and KIN groups (*p* = 0.98). Results showed the KIN + VI group participants demonstrated an improvement in imagery vividness after imagery training (negative change in the % VMIQ-2 score means improvement in the imagery vividness).

Participants’ intensity of using specific imagery modalities during training is reported in Table 5: results show high usage of KIN modality for both groups with no difference in the levels of usage. Nonetheless, results show a higher usage of VI modality in the KIN + VI group than in the KIN group, which is consistent with study instruction (*t* (30) = 3 .554, *p* = 0.001). Furthermore, participants in both intervention groups, who used VI, tended to use an IVI perspective rather than using an EVI perspective. Moreover, the VMIQ-2 results revealed that the imagery capability could be significantly improved by the combination of KIN&VI modalities, while no improvement could be detected in the other study groups (Table 5).

**TABLE 5:** Participant’s compliance data during imagery intervention, and Score of VMIQ-2.

Parameter	KIN + VI (n = 16)				KIN (n = 16)	
KIN usage	7.19 ± 1.91				7.63 ± 1.50	
VI usage	7.44 ± 1.50				5.25 ± 1.95	
VI Perspectives	3.31 ± 2.33				3.75 ± 2.62	
Score of VMIQ-2 subscales study 1						

Parameter	KIN + VI (n = 16)		KIN (n = 16)		CTRL (n = 15)	
	Pre	Post	Pre	Post	Pre	Post
EVI	30.63 ± 9.19	29.00 ± 7.66	28.93 ± 8.96	30.47 ± 9.20	28.27 ± 10.41	28.00 ± 10.66
IVI	28.13 ± 7.02	23.50 ± 6.61*	23.53 ± 7.75	26.60 ± 8.14	23.47 ± 9.55	25.93 ± 8.59
KIN	27.63 ± 9.82	24.63 ± 5.83	27.27 ± 8.39	27.13 ± 7.99	22.93 ± 6.88	26.80 ± 9.57
Total score	86.38 ± 16.58	77.13 ± 12.05*	79.73 ± 18.13	84.20 ± 21.14	74.67 ± 20.92	80.73 ± 23.05

KIN, usage: participants self-report the intensity of using kinaesthetic imagery modality during the imagery training. VI, usage: participants self-report the intensity of using visual imagery modality during the imagery training. VI, perspectives: participants reported intensity of using different visual imagery perspectives (IVI: internal visual imagery or EVI: external visual imagery); VMIQ-2: Vividness of Movement imagery Questionnaire-2; EVI: external visual imagery subscale; IVI: internal visual imagery subscale; KIN: kinaesthetic imagery subscale. Total score: the total score result from summation of three VMIQ-2, subscales. Data are presented as means ± standard deviation.

### Study 2

Data from the weekend logs indicated all participants reported 100% compliance with the assigned home-protocol sessions. However, the independent *t*-test showed a significant difference between study groups on the level of motivation to perform the assigned home-training sessions (*t* (28) = 4.422, *p* = 0.00). Specifically, higher motivation scores were reported in the *Imagery* group (8.50 ± 1.05) compared with the *Exercise* group (6.38 ± 1.53).

Participants’ intensity of using specific imagery modalities in training are reported for the *Imagery* group: results showed high usage of KIN (8.60 ± 0.99) and VI modalities during imagery training (8.13 ± 0.83), which is consistent with our study instructions. Furthermore, results showed all participants in the *Imagery* group, who used visual imagery, tended to use an IVI perspective rather than an EVI perspective or a strategy of switching between perspectives (1.80 ± 1.61).

**FIGURE 4:**
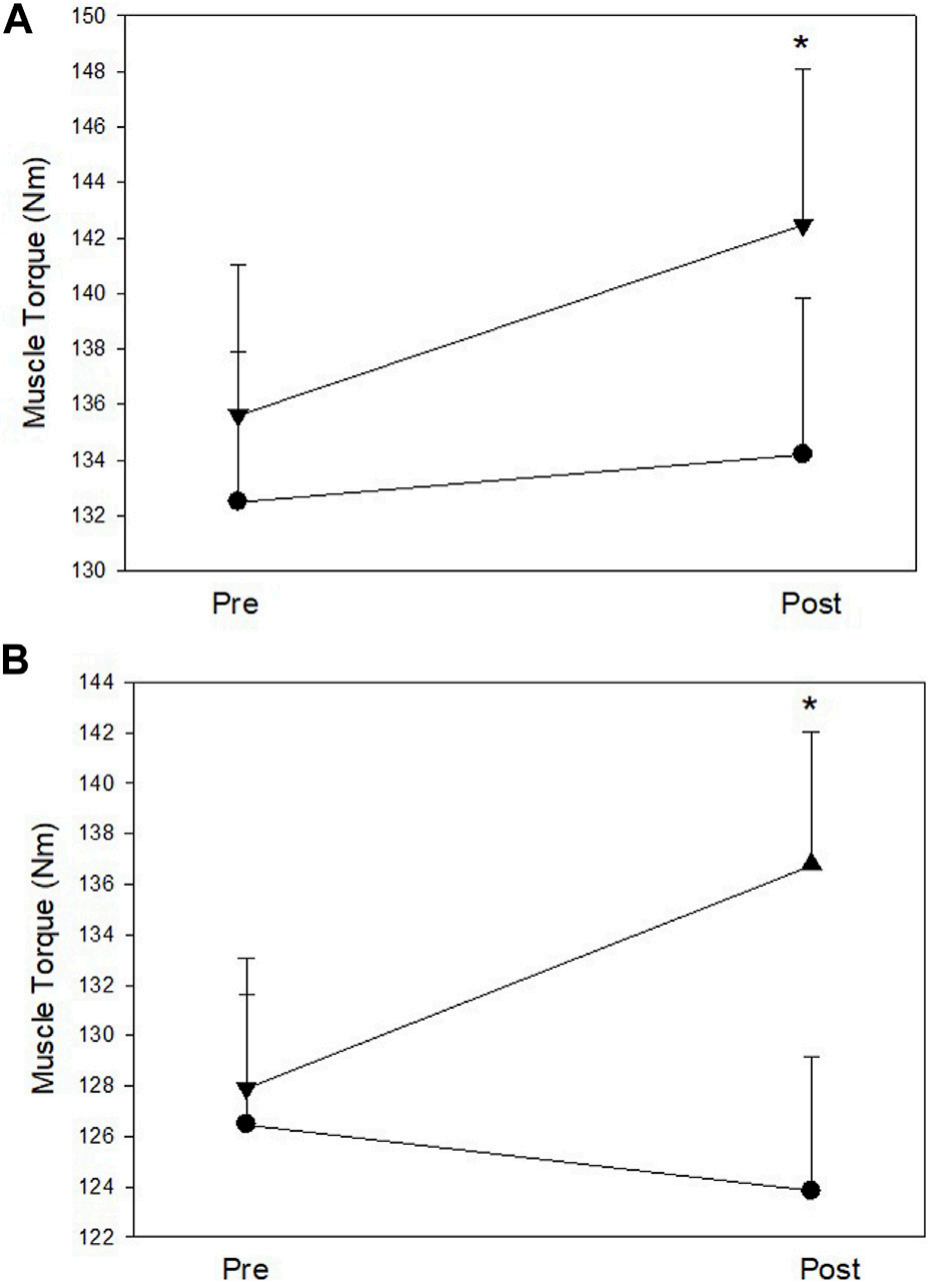
**(A)** Right (trained) hip abductor muscle strength at pre and post assessments; **(B)** Left (untrained) hip abductor muscle strength at pre and post assessments. Filled circles: Exercise group; Filled triangles: KIN + VI group. Mean and standard errors; *significant difference, *p* < 0.05.

The MIT data of the **trained** right hip abductors at pre- and post-test are shown in Table 6 (Figure 4A). Analysis of pre and post intervention periods (mixed model ANOVA) reported a significant increase in MIT of the right hip abductor muscles over time (*F* (1, 28) = 9.29, *p* = 0.005, η^2^ = 0.25), with no significant effect between groups. However, results demonstrated a trend interaction between study groups and MIT alteration over time (*F* (1, 28) = 3.38, *p* = 0.08, and η^2^ = 0.11). Further, within group analysis demonstrated that participants in the *Exercise* group did not show significant MIT alterations over time, while MIT of the *Imagery* group increased significantly (∼7%) (*t* (28) = −4.12, *p* = 0.001).

**TABLE 6:** MIT of hip abductor muscles across both study groups at pre- and post-assessments.

Study groups	MIT (Nm) trained right hip abductor muscles		
	Baseline Strength	Post-Strength	
***Exercise* (n = 15)**	132.51 ± 19.19	134.21 ± 17.71	
***Imagery* (n = 15)**	135.62 ± 25.53	142.48 ± 25.11*	

	MIT (Nm) untrained left hip abductor muscles		
	Baseline Strength	Post-Strength	
***Exercise* (n = 15)**	126.55 ± 17.75	123.85 ± 13.38	
***Imagery* (n = 15)**	127.92 ± 21.92	136.78 ± 25.31*	

Data of paired sample t-test (within group comparison). MIT: Nm: Newton*meter. *, significant difference (*p* < 0.05) between pre and post assessment within each group.

The MIT data of the untrained left hip abductors at pre- and post-test are listed in Table 6 (Figure 4B). Mixed model ANOVA reported a trend increase of the MIT (*F* (1, 28) = 3.19, *p* = 0.085, η^2^ = 0.10), but MIT alterations did not show a significant difference between groups. However, a significant interaction between study groups and MIT alteration over time was found (*F* (1, 28) = 11.25, *p* = 0.002, η^2^ = 0.29). Further within group analysis demonstrated that MIT of the *Imagery* group increased significantly (∼8%) (*t* (28) = −3.21, *p* = 0.006), while no significant MIT alteration of the **untrained** left hip abductor muscles from baseline was found in the *Exercise* group.

MIT data of left-hand grip and right shoulder abductors at pre- and post-test are shown in Table 7. Mixed model ANOVA reported no significant main effects in left hand grip strength alterations over time, or between groups. In addition, results did not show a significant interaction between groups and strength alteration over time (*p* > .05).

**TABLE 7:** Left hand grip strength/MIT of right shoulder abductor muscles across both study groups at pre- and post-assessments.

Study groups	HGS (kg)	
	Pre	Post
***Exercise* (n = 15)**	43.33 ± 7.10	44.39 ± 6.80
***Imagery* (n = 15)**	43.78 ± 6.51	43.55 ± 6.54

	MIT (Nm) Right shoulder abductors	
	Pre	Post
***Exercise* (n = 15)**	69.79 ± 10.50	65.63 ± 11.10
***Imagery* (n = 15)**	64.84 ± 6.51	63.86 ± 7.55

Data of paired sample t-test (within group comparison). HGS: hand grip strength, the maximum isometric strength of the left hand and forearm muscles. kg: Kilograms. MIT: Nm: Newton*meter.

**FIGURE 5:**
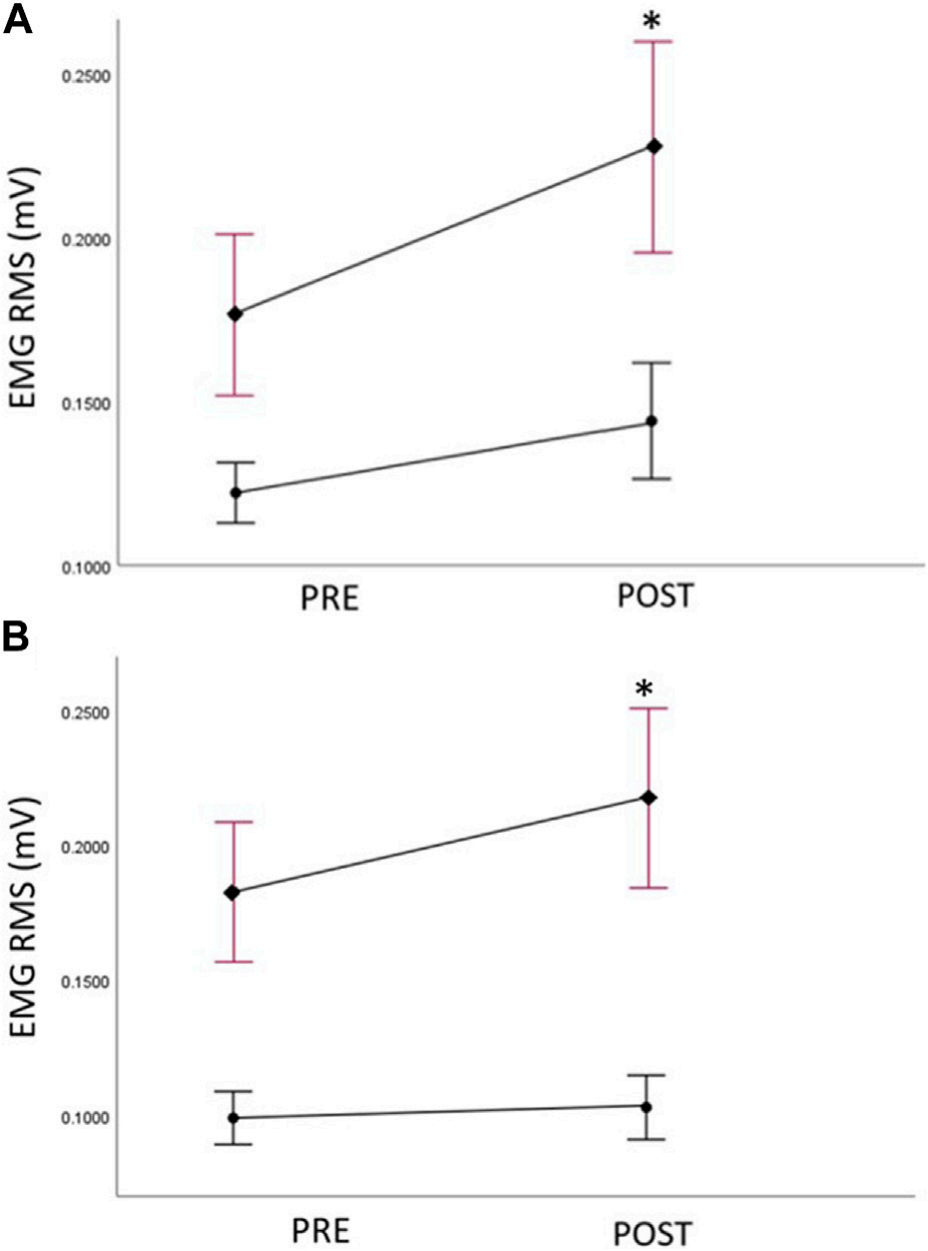
**(A)** Right (trained) hip abductor muscle EMG amplitudes (root mean squared—RMS) at pre and post assessments, filled circles: Exercise group; filled diamonds: KIN + VI group; **(B)** Left (untrained) hip abductor muscle EMG amplitudes (root mean squared—RMS) at pre and post assessments, filled circles: Exercise group; filled diamonds: KIN + VI group. Mean and standard errors; *significant difference, *p* < 0.05.

The EMG data of the GM (both sides) at pre- and post-assessments are reported in Table 8 (Figure 5). At baseline, no significant between group difference of EMG amplitude was detected for the trained right GM; however, a significant between group difference was observed for EMG amplitudes on untrained left (Mann-Whitney U test, *U* = 56, *p* = .014), with lower EMG amplitudes in the *Exercise* group. For the *Imagery* group, a significant increase in the EMG amplitude in the trained right GM was detected following the intervention period (Wilcoxon signed-rank test; Z = −3.24, *p* = 0.001), however, no difference in EMG amplitude for the *Exercise* group was detected. Moreover, the *Imagery* group revealed a significant increase in EMG amplitude in the untrained left GM (Wilcoxon signed-rank test; Z = −2.59, *p* = 0.01), while the *Exercise* group did not reveal a significant change in EMG amplitude between timepoints.

**TABLE 8:** EMG data of GM for both sides at pre- and post-assessments.

		*Exercise group (n = 15)*				*Imagery* group (n = 15)			
		EMG-Amp (mV) hip abductors							
		Trained Right		Untrained Left		Trained Right		Untrained Left	
		Pre	Post	Pre	Post	Pre	Post	Pre	Post
**Mean**		0.122	0.144	0.099	0.103	0.182	0.234	0.185	0.220
**Std. Deviation**		0.036	0.068	0.038	0.046	0.095	0.125	0.100	0.129
**Percentiles**	25th	0.100	0.094	0.067	0.059	0.086	0.096	0.095	0.109
	50th (Median)	0.124	0.135	0.109	0.093	0.159	0.195	0.178	0.209
	75th	0.137	0.206	0.126	0.151	0.270	0.375	0.267	0.349
**Z**		−0.973		−0.596		−3.238		−2.585	
*p* **-value**		0.33		0.55		0.001*		0.010*	

The Wilcoxon signed-rank (within group comparison). EMG-Amp: mV: millivolts. *, significant difference (*p* < 0.05) between pre and post assessment within each group.

Shoulder muscle EMG of the right deltoideus medius (R-DM) revealed no significant difference between study groups (*p* > .05). However, the Wilcoxon signed-rank test reported a significant decline between pre- and post-assessments in the *Exercise* group. In the *Imagery* group, no significant difference in EMG amplitude was found (Table 9).

**TABLE 9:** EMG data of R-DM at pre- and post-assessments.

		*Exercise* group (n = 15)		*Imagery* group (n = 15)	
		EMG-Amp (mV) right deltoideus medius			
		*Exercise* group		*Imagery* group	
		Pre	Post	Pre	Post
**Mean**		0.501	0.419	0.608	0.630
**Std. Deviation**		0.149	0.118	0.366	0.351
**Percentiles**	25th	0.401	0.327	0.389	0.480
	50th (Median)	0.433	0.420	0.542	0.648
	75th	0.666	0.496	0.823	0.819
**Z**		−2.045		−0.057	
*p* **-value**		0.041*		0.955	

The Wilcoxon signed-rank (within group comparison). EMG-Amp: mV: millivolts. *, significant difference (*p* < 0.05) between pre- and post-assessments within each group.

Both groups were assessed for motivation and effort for performing the strength tasks for the hip muscles after pre and post measurement sessions (Table 10). Analysis of motivation changes pre to post the imagery and exercise training revealed no significant difference between the groups (F (1.28) = 1.042, *p* = 0.316, η ^2^ = 0.036). Correlation analysis of motivation changes with strength changes (%) pre to post the interventions across both groups showed that there was a significant low correlation in the imagery/exercise trained hip muscles (R = 0.388, *p* = 0.34) but not in the untrained hip muscles (R = 0.070). Analysis of effort changes pre to post interventions revealed no significant difference between groups (F (1.28) = 0.398, *p* = 0.533, η ^2^ = 0.014). In addition, effort changes pre to post for performing the strength tasks were not significantly correlated across groups with strength changes (%) neither in the imagery/exercise trained hip (R = 0.257), nor in the untrained hip (R = −0.113).

**TABLE 10:** Levels of motivation and effort.

Study groups	Effort score (zero: no effort at all, to 130: Extreme effort)	
	Pre	Post
Exercise (n = 15)	105.5 ± 24.5	106.5 ± 16.5
Imagery (n = 15)	100.7 ± 19.3	109.8 ± 11.1

	Motivation score (zero: not motivated at all, to 10: very highly motivated)	
	Pre	Post
Exercise (n = 15)	8.60 ± 1.30	8.80 ± 1.21
Imagery (n = 15)	8.13 ± 1.60	8.87 ± 1.06

## Discussion

The primary research objective of our first study was to explore the effectiveness of two imagery practice protocols (KIN and a combination of KIN + VI) compared to control on unilateral hip abductors strength (ipsilateral effect). Results showed that imagery training can lead to increased maximal isometric force production in a larger muscle group (i.e., the hip abductors). This was particularly apparent with the use of the combination imagery protocol (kinaesthetic and visual imagery; KIN + VI)) where significant increases in muscle strength (∼8%) were observed.

In the second study, we investigated the efficacy of the combined imagery protocol KIN + VI in comparison with exercise training on bilateral hip abductors strength and EMG amplitude. Results confirmed main findings of the first study with improvement in hip abductors’ strength (∼7%) occurring in the imagery group. In addition, the capability of imagery training for improvement in strength and EMG amplitudes in the contralateral untrained hip abductor muscles revealed a bilateral transfer effect. Moreover, current data showed that the 2 weeks exercise training did not affect strength of the trained hip abductors and did not result in a transfer-effect on the contralateral untrained hip abductor muscles. Results indicating the benefits of combined imagery (KIN and VI) on both ipsilateral and contralateral muscle groups strength development.

### Imagery modalities and perspectives effects

Data from both studies showed that adding the visual modality to the kinaesthetic modality resulted in better muscle strength outcomes (compared to VI alone, control, or physical training). Participants in KIN + VI group reported using mostly the internal visual perspective as opposed to an external visual perspective (based on VMIQ-2 assessment, not shown). In earlier studies, participants were instructed during imagery training (e.g., as a part of the imagery script) to adopt various imagery modalities and perspectives (e.g., visual, kinaesthetic, stimulus and response propositions), while researchers did not instruct participants to adopt specific sensory modalities or perspectives during imagery training (Ranganathan et al., 2004; Reiser, 2011; Yao et al., 2013; Grosprêtre et al., 2018). Accordingly, outcomes often cannot be conclusively linked to a specific imagery modality or technical feature of the training protocol. However, Yao et al. (2013) were the first study to show better efficiency of internal imagery (i.e., defined as a combined KIN + IVI) compared with external imagery (also known as third-person visual imagery) on elbow-flexion muscle strength (Yao et al., 2013). Internal perspective during imagery practice seems to be relevant for achieving better strength outcomes, which is consistent with our findings. However, motor learning in training has been shown consistently to be more successful with external focus of attention (Wulf, 2013; Milley and Ouellette, 2021). Indeed, Halperin et al. (2016) showed that isometric maxima were higher with external focus rather than internal focus, supporting the view that external focus can lead to more automatic motor response and less disruption of automatic control during performance (Halperin et al., 2016). In our exercise protocol, the focus of attention perspective may have been ambiguous for the performer, therefore more internal focus during exercise training might have confounded potential effects of exercise training which could have been achieved by clear external focus of attention.

In both studies, data revealed that a short period of imagery training (2 weeks) can be effective for improvement of muscle strength and imagery vividness (VMIQ-2 total score); this improvement specifically occurred in the internal visual imagery ability subscales.

Consistent with our findings, earlier investigation found improvement in visual movement imagery ability following 16 weeks of imagery training compared with verbalization training (Robin et al., 2007). Imagery ability is an essential factor for the success of imagery intervention, as individuals with higher imagery ability benefit more from imagery interventions compared with individuals with lower ability (Hall et al., 1992; Leung et al., 2013). The authors advocate the use of the current studies imagery interventions as a method to increase imagery ability.

### Muscle strength improvement and potential mechanisms

Yue and Cole (1992) performed one of the first investigations showing the efficacy of imagery training for strength improvement in the finger muscles (Yue and Cole, 1992). Subsequent studies have indicated that imagery training (i.e., with different intervention periods ranging from 4 to 7 weeks) can improve muscle strength in various muscle groups (Paravlic et al., 2018; Liu et al., 2023). Specifically, upper limb muscle groups, comprising the elbow flexor (Herbert et al., 1998; Smith et al., 2003; Shackell and Standing, 2007), dorsal extension and ulnar abduction and fifth finger abductor digiti minimi muscle (Herbert et al., 1998; Smith et al., 2003; Smith and Collins, 2004), and lower limb muscle groups, namely, the plantar flexor (Zijdewind et al., 2003; Bouguetoch et al., 2021), ankle dorsiflexor (Sidaway et al., 2005), and hip flexor (Yao et al., 2013). The previous studies showing improvements in strength/performance outcomes, mostly adopted a cognitive imagery type of intervention. This intervention type comprises mental simulation of the physical task that would be used during muscle strength assessment, without any overt movement. Our results are consistent with these previous studies but using larger muscles and a shorter intervention period (i.e., 2 weeks), which could prove extremely beneficial in the rehabilitation setting.

Improvement of strength in the trained hip abductors was not apparent following 2 weeks isometric exercise training. A factor that may explain the inefficacy of isometric training protocol of our intervention could be related to the short period of training. Minimal isometric training period for reported strength improvements has been 3 weeks (SZETO et al., 1989; Weir et al., 1995). Furthermore, training of the hip abductors with isometric maximal contractions was certainly new to the participants and several participants in the *Exercise* group reported discomfort during maximal contractions in the training sessions. Repetitive aversive perceptions during training might have led to predictive learning of aversive perceptual responses (Ploghaus et al., 2000) in connection with maximal isometric contraction of the hip abductors. Therefore, these effects could have masked the improved motor learning and limited the generation of forces to the maximum. Finally, participants in the *Exercise* group displayed less motivation to perform the weekend sessions than those of the *Imagery* group. Consequently, less motivation for training could have influenced the outcome for strength improvements for the exercise group.

Assessing a potential influence of motivation and effort for performing the strength tasks during pre and post assessments, we found a significant difference in motivation changes pre to post assessments between CTRL and KIN group, with smaller motivation changes in the CTRL group in study 1. However, the influence of motivation changes on strength changes across all groups showed only a low correlation which suggests that the influence on strength outcomes for study 1 is small and apparent in all groups. The motivation levels of the control groups might have been influences by the lack of contact to the research team between pre and post assessments. However, the effort measure did not show any differences between groups and no correlation across groups for study 1. Considering the differences between treatment and control group in motivation in study 1, strength improvements need to be seen with caution in relation to its effect size.

The above limitations regarding the effect of imagery training are, however, not seen in study 2. There were no group differences in changes of motivation as well as effort for performing the strength tasks with the hips muscle on both sides (trained/exercise and without training/exercise), during the assessment sessions. A low correlation was found between motivation changes and strength task performance for the trained hip side, showing that there was an influence of motivation for strength outcomes across the groups which did not confound the overall outcome due to the variability of motivation affected both groups equally.

EMG amplitudes increased following imagery training. This result implies an improved motor command to the muscle groups as a possible reason for strength improvements. To avoid direct training effects on the muscle during imagery training sessions, EMGs were controlled during training and remained almost quiescent. This approach was consistent with a previous study, showing that EMG signals from the major elbow flexor muscle during imagery training sessions remained well below 2% maximal contraction level (Herbert et al., 1998). The absence of EMG activity is required as a precondition to execute a particular imagery task (Ranganathan et al., 2004), which excludes any motor learning process based on afferent feedback from muscle sensors (Herbert et al., 1998; Shackell and Standing, 2007).

In support of EMG alterations during MIT in the imagery group, Ranganathan et al. (2004) found a significant increase in EMG amplitude in the finger abductors (ABD group) and biceps brachii (ELB group) following imagery training for 12 weeks (Ranganathan et al., 2004). EMG amplitudes in finger muscle have been associated with strength changes after imagery training (Smith and Collins, 2004). Here, imagery training outcomes have be explained by central neural adaptation, with increased cortical potential and motor output observed. Elevation in Movement-Related Cortical Potential (MRCP) over both primary motor and supplementary motor cortices suggest that imagery training augments cortical motor output (Herbert et al., 1998; Shackell and Standing, 2007). In addition to the strength improvement in the trained muscles, we also demonstrated that imagery training induced a bilateral transfer effect on contralateral untrained hip abductors (∼8% increase in strength). This effect was not seen in the exercise group. Moreover, a transfer effect was not inducible in other untrained functional analogue muscle groups, such as shoulder abductors, as well as in handgrip strength. These findings suggest that the improvement was not down to a generalized increase in motor command.

Similarly, Yue and Cole (1992) revealed significant muscle strength improvement in the maximal abduction force of the right (untrained) fifth digit muscle following both unilateral imagery and voluntary contraction training (10% and 14%, respectively) in a small muscle group (Yue and Cole, 1992). More recently, Amemiya *et al.* (2010) used a tapping sequence model (performance outcome) to evaluate the occurrence of inter-manual transfer (bilateral effect) after motor imagery and to compare the transfer effects with motor execution learning (Amemiya et al., 2010). Results showed that the motor execution training improved the performance only for trained movements, while the imagery training improved the performance for trained movement and inter-manual transfer. Moreover, Land *et al.* (2016) explored imagery training effects on bilateral transfer compared with motor execution, by examining the influence of practice duration and task difficulty on the extent to which imagery training and physical training influenced a bilateral transfer on a sequential key pressing task (Land et al., 2016). They concluded that imagery training benefits bilateral transfer primarily at the initial stages of learning, but with extended training, physical practice leads to larger influences on transfer.

The potential underlying mechanisms of the bilateral transfer phenomenon has been broadly classified into central and peripheral adaptations (Lee et al., 2009). Regarding bilateral effects of unilateral strength training, Carroll *et al.* (2006) suggested that the bilateral training effect is caused by central mechanisms increasing the motor neuron output (Carroll et al., 2006). They proposed central adaptation involving a “spill over” to the motor control areas for the contralateral limb. They suggested that the cortical, subcortical, and spinal levels are all potentially involved in the “transfer effects”, and that none can be excluded based on the existing evidence. In principle, three models for the bilateral transfer effect are postulated on central levels but mostly tested for motor skills of the hands. Ruddy and Carson, (2013) postulated that neural adaptations induced during unilateral exercise would spread to the opposite side of the body (cross-activation model; (Parlow and Kinsbourne, 1989; Ruddy and Carson, 2013). At sub-cortical and cortical levels, previous work confirms the presence of a neural interaction between the two hemispheres (Carroll et al., 2006; Farthing et al., 2007), supporting the cross-activation/spill over model proposed by (Carroll et al., 2006; Ruddy and Carson, 2013). Secondly, that the trained motor plan of a unilateral task is accessible by an attempt of reproducing the same task in the opposite side of the body and would therefore facilitate motor activation in the untrained limb (access model/callosal model; (Sainburg and Wang, 2002)). The motor ability, strength task in our study, generated in the trained hemisphere, would reach to the opposite side through the corpus callosum facilitating the task performance in the untrained side with increasing strength generation. A third model proposes that motor programs are established through training in both sides, therefore, also contra-lateral to the trained side, leading to task improvement in the untrained side (Chase and Seidler, 2008). In addition, a recent study suggested that the bilateral transfer effect may also involve the mirror neuron system possibly contributing to motor learning by creating a motor image via interaction with anticipatory motor areas and dorsolateral prefrontal cortex (Uggetti et al., 2016).

The bilateral transfer effect in the current study did not occur in other muscle segments, i.e., no strength gains in the untrained muscle groups in upper limb segments (shoulder abductors) and handgrip were found following either exercise or imagery training of the hip abductors. Hence, our results suggest that our imagery-training effects are specifically improving the trained hip abductors strength and that the effects on the contralateral untrained hip abductor muscles is motor plan specific. If the imagery training would facilitate general motor output for tasks, shoulder and hand grip performance improvements would have been detected. However, our experiments cannot differentiate between the potential mechanisms involved in the bilateral transfer effect, i.e., preference of cross-activation or access model.

The study has several limitations; both studies involved healthy participants who were physically active. This may have left little room for strength enhancement following imagery training. Thus, further investigation needs to be performed, particularly within clinical populations. The intervention protocols were short and therefore it is not clear whether longer protocols would have led to strength gains in the exercise group; the superiority of imagery training over the exercise training on strength gains might be limited to short training protocols (Yue and Cole, 1992; Farthing et al., 2007).

In addition, the participants in study 1 received reimbursement for their time as recommended by Breitkopf et al., (Breitkopf et al., 2011), which resulted in uneven pay after the study. Participants, however, were not aware of the difference in final pay until after the study finished. However, predictions by the individuals could have influenced motivation for performing tasks, which was, however, assessed in the study.

In summary, current findings show that imagery training has clear effects on strength and EMG gains in trained and untrained lower limb muscle groups without negative side effects. Imagery-based intervention are cost effective and easy to apply and may be appropriate for use with diverse medical conditions. Thus, the current findings suggest that motor imagery, more specifically KIN and VI combined, could be used at different stages of rehabilitation with varied patient groups (e.g., injured athletes, neurological or musculoskeletal conditions) to improve strength and motor function. This is particularly beneficial when the use of therapeutic exercise is limited due to injury, surgery, pain, immobilisation, or time and access constraints.

## Data Availability

The original contributions presented in the study are publicly available. This data can be found here: https://doi.org/10.5281/zenodo.8233969.
