# Supplementary material for: Influence of motor imagery training on hip abductor muscle strength and bilateral transfer effect
Source: Front Physiol. 2023 Sep 7;14:1188658. doi: 10.3389/fphys.2023.1188658 (PMC10512955; doi:10.3389/fphys.2023.1188658)
Supplement: Supplementary file 1 [file DataSheet1.PDF]

Supplementary Information

Protocols

S1. Intervention protocols combined with home protocol

The exercise training

Participants received an explanation, demonstration, and were familiarized with the physical practice. The demonstration, and familiarization enabled the participant to perform the exercise in a standardized manner under the experimenter's control, as well as at home. The following instruction has been delivered to the participant by the researcher for this practice:

- Participants have been instructed to lie down on their left side on the dynamometer table, and their right leg was in the top position. This enabled them to prepare themselves for the physical practice of right hip abduction.
- Participants had a full explanation of the exercise practice, and one session of max isometric contractions as part of the pre-test assessments (earlier visit) and to secure that participants can perform physical practice with the protocol below.

- Figure: Volunteering participant (image used with permission) for measurement of contractions and electromyogram of the gluteus medius
- 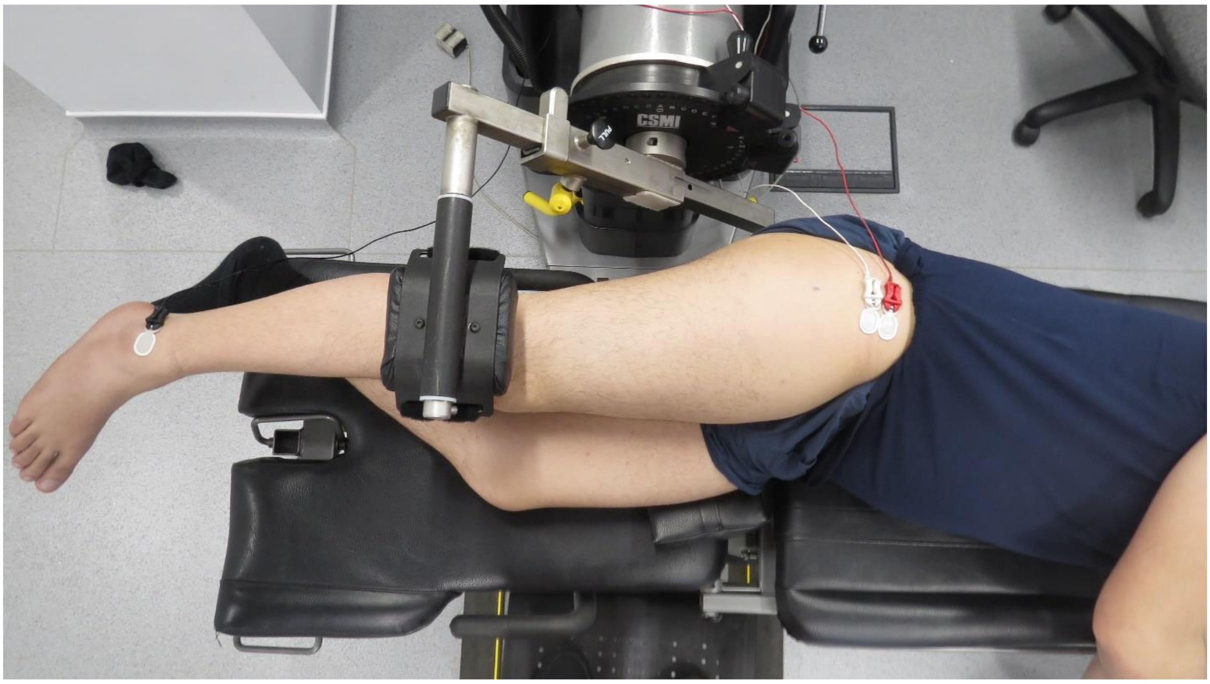

The physical training practice:

- Participants were positioned on the dynamometer table and connected to equipment, as it has been for the pre-test assessment (Figure). Participants performed maximal isometric exercises. The right leg has been elevated

towards the dynamometer arm to perform maximal isometric hip abduction exercise at 25-degree angle.

- Participant were instructed to perform maximal physical contractions of right hip abduction by the following verbal instruction; **please note:** in the home sessions participant were asked to perform the exercise without additional loading, just to elevate the leg and hold it at the 25-degree position matching the pattern of supervised exercise.
- **Starting position: "**Lie on your left side, with your head supported, and both knees straight and feet together. Elevate your leg until you feel the solid resistance of the dynamometer arm – this is at 25˚ of hip abduction."
- **Action:** "Keep your leg in the former position 25˚of of hip abduction, and then slowly push your right leg in abduction direction against the dynamometer lever as much as you can (MAXIMAL FORCE!). Keep pushing the dynamometer arm as much you can for 15 seconds (timed by the experimenter).
- Then slowly relax your hip muscles and move your leg down into starting position (legs on top of each other with feet together."
- **Key points:** "Ensure your pelvis does not rotate backward during pushing."

- The participants performed the physical exercise practice of maximal isometric contractions of hip abduction with the pattern below. EMG electrodes were connected to the right gluteus medius muscle during the second training session to record the muscle activation during the physical exercise. Further training sessions were carried out without EMG recordings except for pre- and post-assessments sessions.
- The training protocol involved 10 daily sessions over two weeks. Each session lasted 30 minutes, including 7 sets of physical exercise training practice. Each set included performing five maximal physical isometric contractions of hip abduction movement each held for ~15 s against dynamometer arm that fixed at 25˚of hip abduction followed by 15s rest.
  The experimenter timed the contraction and relaxation exercises, telling when to start and finish. Consequently, participants performed seven sets of physical exercise training during each session, and each set was followed by maximal 2 minutes rest. A total of 35 maximal physical isometric contractions of hip abduction were performed during each session.
- **Home-protocol**: participants performed one home session each day on the weekends and record their motivation on a diary sheet; they performed 7 sets of physical training practice and each set includes performing five isometric contractions of hip abduction movement each held for ~15 s at 25˚of hip abduction followed by 15s rest. The only difference between the home and lab sessions was that participants were asked to perform an unloaded exercise, just elevating and holding the leg matching the pattern of supervised exercise. The participants were provided with a stopwatch for performing the exercise at home.

Overview of the session:

**1st set of contractions**<br>1 practice with conducting under experimenter guidance<br>5 physical isometric contractions for 15 seconds each (timed by the experimenter.)<br>**Rest** up to 2 minutes
**2nd set**<br>5 physical isometric contractions for 15 seconds each (timed by the experimenter.)<br>**Rest** up to 2 minutes
**3rd set**<br>5 physical isometric contractions for 15 seconds each (timed by the experimenter.)<br>**Rest** up to 2 minutes
**4th set**<br>5 physical isometric contractions for 15 seconds each (timed by the experimenter.)<br>**Rest** up to 2 minutes
**5th set**<br>5 physical isometric contractions for 15 seconds each (timed by the experimenter.)<br>**Rest** up to 2 minutes
**6th set**<br>5 physical isometric contractions for 15 seconds each (timed by the experimenter.)<br>**Rest** up to 2 minutes
**7th set**<br>5 physical isometric contractions for 15 seconds each (timed by the experimenter.)<br>**Rest** up to 2 minutes --

End of session

Imagery training protocol

The participants received an explanation and demonstration of the imagery practice. The demonstration enabled the participant to perform the imagery practice in a standardized manner under the experimenter's control, as well as at home. The following instruction were delivered to the participant by the researcher for the practice:

- Participants were instructed to lie down on their left side on the dynamometer table, and their right leg positioned on top of the left with feet together, legs stretched out. This enabled them to prepare for the imagery practice of right hip abduction.

- Participants of the imagery group have had a full explanation of the physical exercise practice, and one session of maximal isometric contractions as part of the pre-test assessments (earlier visit) and to secure that participants could perform imagery practice with the protocol below.

The imagery training practice structure:

- The participant was positioned on the dynamometer table and connected to equipment, as it had been for the pre-test assessment performing maximal isometric exercises. The right leg was on the top of the left leg, feet together. However, it has been rested without any hip abduction to enable the participant to perform the imagined isometric hip abduction exercises from this position without any actual physical contractions.
- Then participant listened to a recording of the mental imagery script. The recording described exactly what the participant should imagine as in the practice session being performed for the imagined contractions. Participants were told to listen to the recording and to perform imagined isometric contractions of hip abduction movements along with the audio recording. The audio recording had instructed the participant to use combined kinaesthetic and visual imagery training of hip abduction without actual contraction of the muscle. **Please note:** the recording referred to the lateral side of the thigh: "This is the side of the right leg that is facing the ceiling when you lying on your left side."

The following part is the imagery script delivered by the audio recording.

- ***"Close your eyes.***
  - Take in a deep breath and prepare your mind to focus on the imagery practice.
  - Now, imagine while you lying on your side on the isokinetic machine, think back to the sensations you felt and your visual image as you performed the maximal contraction of your thigh muscle.
  - Feel and see your top leg rise to meet the fixed dynamometer arm. Feel and see the cushion of the dynamometer touch the side of your thigh just laterally above the knee. Feel and see the muscles on the lateral side of your thigh, from your hip down to your knee, begin to tense up. Feel and see your thigh pushing against the fixed dynamometer arm.
  - Now, feel and see the pad of the dynamometer arm pressing harder and harder into the lateral portion of the thigh. In other words, try to retrieve your feeling and visualization of the same position, muscle contractions, and action that you experienced when your hip abductor was measured by the isokinetic machine.
  - Feel and see your muscles contract more and more for 15 seconds. <reader pauses for 15 seconds>
  - Now, feel and see your thigh muscles begin to relax. Feel and see the cushion of the dynamometer pressing against your thigh less and less, and bring your leg back to the neutral position."

- <participant gets a break for 15 seconds>

- Next, participants performed a further imagery practice of maximal imagined contraction of hip abduction. The EMG electrodes were connected to the right gluteus medius muscle during one of the imagery sessions (session 2) and on pre-and post-assessment visits for recording muscle activation during the various conditions.
- The training protocol involved 10 daily sessions over two weeks, each session lasted 30 minutes, including 7 sets of imagery training practice. Each set included listening to imagery script instruction while performing the imagined isometric contractions as explained above. In each set five maximal imagined isometric contractions of hip abduction muscles for a duration of ~15 s were performed, followed by 15s rest. The experimenter had been timing those and told participants when to start and finish. Therefore, participants during each session performed 7 sets of imaginary training. A total of 35 maximal imagined isometric contractions of hip abductions were performed during each session.
- **Home-protocol**: participants performed one home session on each day of the weekends and recorded their motivation on a diary sheet. They performed 7 sets of imagery training practice and each set included performing five maximal imagined isometric contractions of hip abduction movement, each for ~15 s followed by 15s rest. The only difference in the home sessions was that participants had been asked to listen to the audio imagery script themselves and to perform imagery practice. In addition, they needed to record the imagery sessions they had performed on the weekends on a diary sheet.

Summary of the session:

**Listen to recording; conduct the imagery as you listen**<br>1 practice with conducting the imagery<br>5 imagined contractions for 15 seconds each (timed by the experimenter.)
**Listen to recording; conduct the imagery as you listen**<br>5 imagined contractions for 15 seconds each (timed by the experimenter.)
**Listen to recording; conduct the imagery as you listen**<br>5 imagined contractions for 15 seconds each (timed by the experimenter.)
**Listen to recording; conduct the imagery as you listen**<br>5 imagined contractions for 15 seconds each (timed by the experimenter.)
**Listen to recording; conduct the imagery as you listen**<br>5 imagined contractions for 15 seconds each (timed by the experimenter.)

**Listen to recording; conduct the imagery as you listen**<br>5 imagined contractions for 15 seconds each (timed by the experimenter.)
**Listen to recording; conduct the imagery as you listen**<br>5 imagined contractions for 15 seconds each (timed by the experimenter.)

End of session
